# Supplementary material for: SIRT7 Deficiency Protects against Aging-Associated Glucose Intolerance and Extends Lifespan in Male Mice
Source: Cells. 2022 Nov 15;11(22):3609. doi: 10.3390/cells11223609 (PMC9688483; doi:10.3390/cells11223609)
Supplement: Supplementary file 1 [file cells-11-03609-s001.zip › cells-1946486-supplementary.pdf]

Supplementary Table S1: Primer sequences of mouse genes used for qRT-PCR.

Symbol	Forward	Reverse
Atf4	TCGATGCTCTGTTTCGAATG	GGCAACCTGGTCGACTTTTA
Calr	GACTGGGATGAACGAGCCAA	GGTTTCCACTCGCCCTTGTA
Canx	GGCTAGACGACGAACCTGAG	AGGCTTCCATTTGCCCTTAT
Cdkn2a	CCAAGAGCGGGGACATCAAG	AAGCTATGCCCGTCGGTCTG
Col3a1	TACACCTGCTCCTGTGCTTC	CATTCCTCCCACTCCAGACT
Col6a1	TGATGGCATTCAAGGACCCC	TGGGCCAGCATTTCCTTCAT
Cxcl2	CCAGACAGAAGTCATAGCCACTC	CTTCCGTTGAGGGACAGCA
Cycs	GGAGGCAAGCATAAGACTGG	TCCATCAGGGTATCCTCTCC
Ddit3	ATATCTCATCCCCAGGAAACG	TCTTCCTTGCTCTTCCTCCTC
Ddost	CAGCGATGCCTTCTTCAACTC	GCCTGTCTGAGAATACCTCTGC
Dio2	AGTCAAGAAGGTGGCATTCG	ACAGCTTCCTCCTAGATGCCT
Fbxo6	CCGTGTGCTGAAGAGAACC	GTCTCCACCTTCCAGCGAT
Fgf21	GCCATTCACTTTGCCTGAGC	ATCCATTCCATCAGGGCTGC
Hspa5	AGTGGTGGCCACTAATGGAG	TCAATCCTTGCTTGATGCTG
Il1b	GGGCTGCTTCCAAACCTTTGACC	GTAGCTGCCACAGCTTCTCCACAGCC
Il4	CATGGGAAAACTCCATGCTT	ATGAATCCAGGCATCGAAAA
Il12a	TGGCTACTAGAGAGACTTCTTCCACAA	GCACAGGGTCATCATCAAAGAC
Lipe	GGCTCACAGTTACCATCTCACC	GAGTACCTTGCTGTCCTGTCC
Pdia3	TGTTGGAACTGACGGACGAA	GGCGAAGAACTCGACTAGCA
Pdia5	TATTCCATTACCAGGACGGCG	TCCTTCTCACTGTCGATGTGC
Pdia6	TGGTTCCTTTCCTACCATCACT	ACTTTCACTGCTGGAAAACTGC
Pnpla2	TCCGAGAGATGTGCAAACAG	CTCCAGCGGCAGAGTATAGG
Ppargc1a	GAAATCCGAGCGGAGCTGAA	GAATAGGGCTGCGTGCCATC
Ppib	AACCACAGTCAAGACCTCC	TCCGTACCACATCCATGC
Sdf2l1	GCTATCCAACAACCAGGAGGTG	GTTCACCAGTGACCGACAGG
Sirt7	TGCCAGGCACTTGGTTGTCT	TAGGCTCCGCTTCGCTTAGG
Slc2a1	GCTTATGGGCTTCTCCAAACT	GGTGACACCTCTCCCACATAC
Slc2a4	ACCCCTCATTCCCCCTGTGT	ACCCTCCTGCAGACCCCTTC
Stt3a	GCAACCTGTCTGATGCTCGGAT	ATGTGGACAGCACCTGGGAAAC
Tbp	CCCCTTGTACCCTTCACCAAT	GAAGCTGCGGTACAATTCCAG
Tnf	AGAAAGCATGATCCGCGACGTG	TGAGAGGGAGGCCATTTGGGAAC
Ucp1	GGCAACAAGAGCTGACAGTAAAT	GGCCCTTGTAAACAACAAAATAC
